# *Panax ginseng* therapy for chronic obstructive pulmonary disease: a clinical trial protocol and pilot study

**DOI:** 10.1186/1749-8546-9-20

**Published:** 2014-08-14

**Authors:** Lei Wu, Anthony Lin Zhang, Yuan Ming Di, Johannah Linda Shergis, Yuanbin Chen, Xinfeng Guo, Zehuai Wen, Francis Thien, Christopher Worsnop, Lin Lin, Charlie Changli Xue

**Affiliations:** 1 Guangdong Provincial Hospital of Chinese Medicine, Guangdong 510120, China; 2 Traditional and Complementary Medicine Program, School of Health Sciences and Health Innovations Research Institute (HIRi), RMIT University, PO Box 71, Bundoora, Victoria 3083, Australia; 3 Department of Respiratory Medicine, Box Hill Hospital and Monash University, Box Hill, Victoria 3128, Australia; 4 Department of Respiratory and Sleep Medicine, Austin Hospital, Heidelberg, Victoria 3081, Australia

## Abstract

**Background:**

*Panax ginseng* (*Ren shen*) has been used to treat chronic obstructive pulmonary disease (COPD). This article aims to present a study protocol and pilot trial comparing *P. ginseng* with placebo for treating moderate to very severe COPD.

**Methods:**

COPD was diagnosed spirometrically, with participants having a forced expiratory volume in one second (FEV1) of between 20% and 79% and FEV1 to forced vital capacity (FVC) ratio of less than 70%. Outcome measures included exacerbation rate, St. Georges Respiratory Questionnaire, COPD Assessment Test and Short-form Health Survey (SF-36). Other outcome measures included the six-minute walk test, FEV1, FVC, relief medication use, use of COPD-specific medical resources, and adverse events. The study is a randomized, double-blind, placebo controlled clinical trial. The method of this pilot trial was based on a planned full-scale trial except that participants were enrolled for ten weeks compared to 52 weeks. In the pilot trial, 14 participants (57–73 years old) with moderate to very severe COPD were recruited from a community health program at a public Chinese medicine hospital in Guangdong Province, China. After a 2-week run-in period, 10 participants were eligible for the study and were randomly assigned to either *P. ginseng* group (n = 5) (200 mg twice daily for four weeks) or placebo group (n = 5), and then followed-up for an additional 4 weeks for a total of 10 weeks.

**Results:**

Nine participants completed the trial and one dropped out. The exacerbation rate could not be evaluated because there were no exacerbations. One participant in *P. ginseng* group reported events of sore throat, cough and fever. Trial investigators did not consider these events as COPD exacerbations or adverse events.

**Conclusions:**

Participant recruitment, study design, data collection and outcome measurement have been tested in a pilot trial. A full-scale trial is warranted.

## Background

Chronic obstructive pulmonary disease (COPD) is characterized by breathlessness, coughing, and excess sputum production [1]. There are 64 million people suffering from COPD worldwide and COPD is predicted to be the third leading cause of death by 2030 [2].

COPD patients often use inhaled bronchodilators to relieve symptoms, and inhaled glucocorticoids to treat and prevent frequent exacerbations [1]. New treatments targeting the underlying chronic inflammatory pathways are being developed [3]. A phosphodiesterase-4 (PDE-4) inhibitor, roflumilast (trade names Daxas/Dalisresp), is used to treat COPD, although it has adverse effects, such as nausea, reduced appetite and abdominal pain [1].

Several systematic reviews indicated *P. ginseng* improved lung function (forced expiratory volume in one second, FEV1) and quality of life measured by the St. George's Respiratory Questionnaire (SGRQ), compared to pharmacotherapy alone [4,5]. Studies investigating *P. ginseng* for COPD and other related diseases such as chronic bronchitis are limited but promising [6-8]. Gross and colleagues demonstrated that *P. ginseng* (G115, 100 mg) twice daily for 12 weeks improved pulmonary function tests, and respiratory endurance in 92 moderate COPD participants [6]. Scaglione and colleagues evaluated *P. ginseng* in two studies. The first involved 40 chronic bronchitis participants and found that *P. ginseng* (100 mg) twice daily for 8 weeks had fewer alveolar macrophages in bronchoalveolar lavage fluid than those who took placebo [7]. The second study involved 75 participants with acute exacerbation of chronic bronchitis and found that those who took *P. ginseng* (G115, 100 mg) twice daily with antibiotics for 9 days had a lower bacterial count than those who took antibiotics alone [8]. In addition, no severe side effect was reported in the *P. ginseng* treatment [9]. Despite positive results, these studies did not evaluate appropriate COPD outcomes, such as health-related quality of life and rates of exacerbation.

We previously designed randomized clinical trials (RCT) to evaluate *P. ginseng*’s effectiveness in treating moderate COPD [10] and identified methods to integrate Chinese medicine in hospital-based clinical trials [11]. This study aims to present the protocol and a pilot trial comparing *P. ginseng* with placebo for moderate to very severe COPD participants.

## Method

### Participants

We included participants who were aged between 40 and 80 years; had a post-bronchodilator FEV1 of ≤ 20% and < 80% of predicted normal values and had a FEV1 to forced vital capacity (FVC) ratio (FER) of less than 70%; were clinically stable and had not experienced an acute exacerbation of COPD for at least 4 weeks before the trial and had not been hospitalised in the past six months with three or more exacerbations; and met the Chinese medicine diagnostic criteria for *lung qi deficiency* with or without *spleen* or *kidney qi deficiency*.

We excluded participants who had a history of asthma or chronic systemic infections or inflammatory conditions other than COPD that require systemic corticosteroid treatment in the last 3 months; were pregnant, breast-feeding or intending to become pregnant during the course of the study; had a serious illness such as severe heart, liver or kidney disease; were taking long-term immunosuppressive agents or immune-stimulants; had an allergic history to ginseng or currently were taking ginseng; were users of monoamine oxidase inhibitor antidepressants, anticoagulants and/or antihyperglycaemic medications; and had undertaken pulmonary rehabilitation within three months of the commencement of the study or intended to enter pulmonary rehabilitation during the study.

### Design

This pilot trial was designed to test the practicality of a full-scale trial that was planned to be conducted in a public hospital in China. The only difference between the pilot trial and the full-scale trial is the trial duration, being 10 weeks for the pilot and one year for the full scale trial.

**Figure 1:**
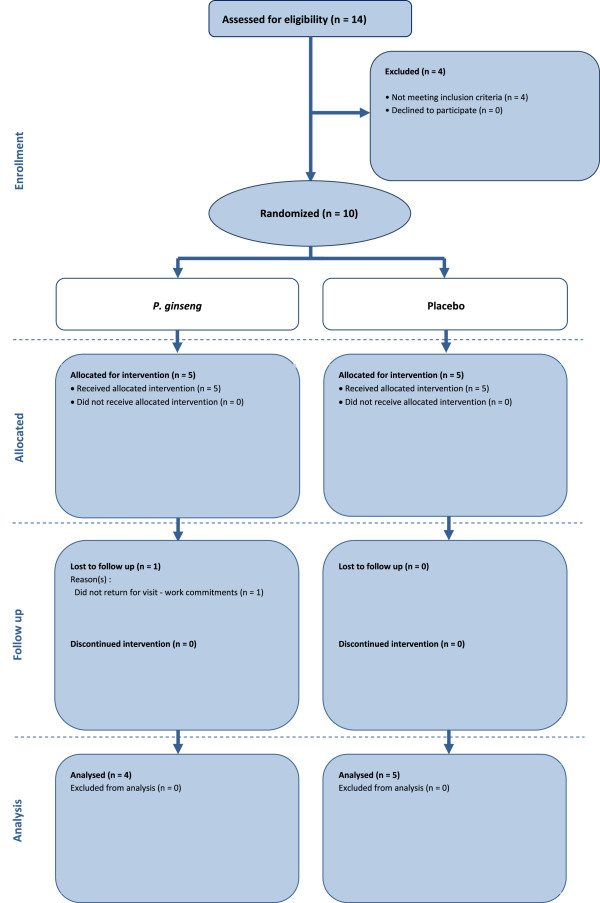
Flow chart describing study design and participant selection in the pilot trial.

**Figure 2:**
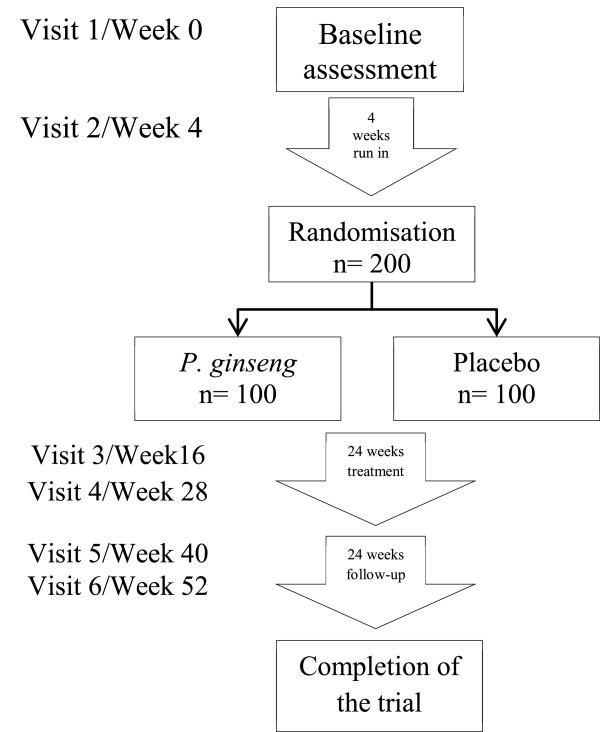
Flow chart for a full-scale trial.

The participants with moderate, severe or very severe COPD (Global Initiative for Chronic Obstructive Lung Disease [GOLD], stage II-IV) [1] were recruited from the Guangdong Provincial Hospital of Chinese Medicine, Guangdong Province, China. The participants who provided informed consent were enrolled for 10 weeks: 2 weeks for run-in; 4 weeks for treatment; and 4 weeks for follow-up. The participants meeting all the criteria entered a 2-week run in period. They were randomized to *P. ginseng* or placebo if they were stable and had no COPD exacerbation during the 2-week run in period. Visits were scheduled for baseline, end of run-in (week 2), end of treatment (week 6) and end of follow-up (week 10) (Figure 1). In the full-scale trial participants will be enrolled for 52 weeks, four weeks run-in, 24 weeks treatment and 24 weeks follow-up (Figure 2). They will attend 6 visits: baseline, randomisation, mid-treatment, end of treatment, mid follow-up and end of follow-up.

Ethical approval (B2012-49-01) was obtained from the Guangdong Provincial Hospital of Chinese Medicine Human Research Ethics Committee (Additional files 1 and 2). The pilot trial has been registered with the Australian and New Zealand Clinical Trials Registry (ACTRN: 12614000029695).

### Random assignment and blinding

A list of randomisation numbers was generated by a statistician using statistical software (SPSS, Windows Version 20.0; IBM Corp., Armonk, NY). This process tested the effectiveness of keeping participants, investigators, medical staff, and other staff blinded to the study allocation. Opaque envelopes containing a number concealed to the treatment allocation was used to randomly assign participants to either *P. ginseng* or placebo group. Medications were put in packages that concealed the randomization code and were dispensed from the hospital’s central pharmacy by pharmacists who were blinded to allocation.

### Interventions

Participants in the intervention group received standardised *P. ginseng* extract, G115, in capsule form 200 mg twice daily and participants in the control group received placebo for 4 weeks. *P. ginseng* G115 is manufactured according to Good Manufacturing Practices by Ginsana SA, Switzerland. The lactose-based placebo was also manufactured by Ginsana SA and matched in appearance, taste and odour. In the full-scale trial medications would be dispensed for 24 weeks.

*P. ginseng*, G115, is the highest quality standardised extract and has been evaluated in more than 46 clinical studies over 35 years [12]. G115 is standardised to contain 4% ginsenosides, Rg1, Rb1, Re, Rf, Rg2, Rc, Rb2, and Rd. The dosage in this study was determined by referencing the clinical trial literature and recommendations from the manufacturer [13].

Throughout the study, participants were given the short-acting β2-agonist Ventolin (salbutamol) to relieve symptoms as needed. Respiratory drugs, including long-acting anticholinergics or long-acting β2-agonists alone or in combination with glucocorticoids, could be used throughout the study under the advice of the participants’ respiratory physicians. Other respiratory drugs, such as short-acting anticholinergics, short-acting β2-agonists other than salbutamol, theophylline, corticosteroids as monotherapy, antibiotics, mucolytics and antitussives were not allowed during the study. The following medications for other conditions were also not allowed during the study: immunotherapy, monoamine oxidase inhibitor antidepressants, anticoagulants, antihyperglycaemics, and other Chinese herbal medicines.

### Outcome measures

The primary outcome measure was rate of exacerbation, which was defined as a change in baseline dyspnoea, cough and/or sputum [14]. Specifically, exacerbations involving two or more symptoms, such as worsening dyspnoea and an increase in sputum purulence or volume or both, or any single major symptom and more than one minor symptom such as upper airway infection, unexpected fever or increased wheezing that lasted two or more days [14]. Investigators (who are qualified respiratory research assistants) phoned the participants at the end of every week and reviewed their participant diary at each visit to determine if a participant had experienced an exacerbation. Investigators prescribed medications to treat exacerbations if needed. Exacerbation severity was categorised as mild (easily tolerated by participant, causing minimal discomfort), moderate (discomfort significant enough to interfere with daily activities) or severe (incapacitating, unable to work or perform daily activities). Exacerbations were not considered adverse events unless they were serious (*e.g.*, fatal, life threatening, permanently incapacitating or left participants needing prolonged hospitalisation).

Secondary outcomes were health status measured with the St. Georges Respiratory Questionnaire (SGRQ), COPD Assessment Test (CAT), the Short-form Health survey (SF-36) and exercise tolerance using the 6-minute walking test (6MWT). Other outcomes included change in postbronchodilator FEV1 and FVC, use of relief medication, and COPD-specific medical resource use, including emergency department presentations and medical practitioner visits. Safety assessments included investigator inquiries about the occurrence of adverse events and blood samples, which included a full blood count and liver/kidney function tests taken before and after treatment.

All outcome measures were collected by the same investigator for continuity and to aid in maintaining a standard procedure. Outcome measures were selected based on the anticipated full-scale trial. For a full-scale trial the sample size and adequate statistical power would be based on a study evaluating carbocisteine for acute exacerbations of COPD [15].

### Statistical analysis

Outcome measures were analysed by *t* test at the end of each time point. SPSS, Windows Version 20.0 (IBM Corp., Armonk, NY) was used to analyse the data for the outcome measures. Last Observation Carried Forward (LOCF) was used to evaluate data with an intention-to-treat analysis. Data were presented as mean and standard deviation. The aim of this pilot trial was to test the practicality of trial design, it would be inappropriate to make any inferences or state findings of any efficacy. Data were only analysed to make the investigators aware of any issues that needed to be overcome in the planned larger-scale trial.

### Results of the pilot trial

We recruited 14 participants from a community health program at the Guangdong Provincial Hospital of Chinese Medicine in May and June of 2012. After the 2-week run in period, 10 of these participants were eligible for the study and were randomly assigned to *P. ginseng* group (n = 5) (200 mg twice daily for four weeks) or placebo group (n = 5) (Figure 1). The four excluded participants did not meet the inclusion criteria due to their lung function and an exacerbation of COPD symptoms during the run-in period and abnormal liver function and a history of liver disease. The participants’ mean age was 64.8 years (range 57 to 73 years old). Nine participants were male and one was female. One participant in the *P. ginseng* group dropped out at week three because of work commitments. Nine participants were classified as having severe COPD, one was classified as having moderate COPD and none were classified as having very severe COPD.

A qualified Chinese medicine respiratory specialist diagnosed the participants’ health according to Chinese medicine. Seven participants were considered to have *lung* and *kidney qi deficiency*, and three participants were considered to have *lung*, *spleen* and *kidney qi deficiency*. At the start of the study, two participants were taking antibiotics, eight were taking long-acting β-agonists, and nine were taking inhaled corticosteroids. Because of the small sample size, several baseline factors, including smoking status and coughing severity, were imbalanced (Table 1).

**Table 1:** Baseline characteristics of the participants

**Baseline assessment**	**Treatment group (n = 5)**			**Placebo group (n = 5)**		
Age (years)	67 ± 4			62.6 ± 4.15		
Male/Female	5/0			4/1		
Smoking status						
Current smoker	3			0		
Former smoker	1			5		
Never smoked	1			0		
FEV1 (L) (post bronchodilator)	1.38 ± 0.63			0.83 ± 0.10		
FVC (L) (post bronchodilator)	2.79 ± 0.59			2.33 ± 0.36		
FEV1/FVC (post bronchodilator)	0.48 ± 0.13			0.36 ± 0.06		
*COPD severity*						
Moderate	1			0		
Severe	4			5		
Symptom severity	Mild	Mod	Sev	Mild	Mod	Sev
Shortness of breath	3	2	0	3	2	0
Cough	2	3	0	5	0	0
Sputum production	3	1	1	5	0	0
Antibiotics	0			2		
Long-acting beta-agonists	3			5		
Inhaled corticosteroids	3			5		
Chinese medicine diagnosis						
*Lung* and *kidney qi deficiency*	4			3		
*Lung*, *spleen* and *kidney qi deficiency*	1			2		

None of the participants experienced an exacerbation during the short study duration (10 weeks). Health status questionnaires (SGRQ, SF-36, and CAT) were unchanged in both groups (see Table 2). All participants, except the one who dropped out, completed the questionnaires and the result was evaluated by LOCF. The baseline questionnaire scores, except those for the SF-36 questions 2 and 3b, 3c, and 3e, were balanced between groups. There was no significant difference in responses to the questionnaires between groups at the end of treatment (see Table 2).

**Table 2:** Outcome measures

	***P. ginseng***		**Placebo**		
	**(mean ± standard deviation)**		**(mean ± standard deviation)**		***P *** **value^**
	**Baseline**	**End of treatment**	**Baseline**	**End of treatment**	
Exacerbations	Not applicable	0	Not applicable	0	1
FEV1 (L)	1.38 ± 0.63	1.40 ± 0.60	0.83 ± 0.10	0.80 ± 0.06	0.06
FVC (L)	2.79 ± 0.59	3.00 ± 0.50	2.33 ± 0.36	2.40 ± 0.46	0.11
FEV1/FVC	0.48 ± 0.13	0.40 ± 0.10	0.36 ± 0.06	0.30 ± 0.06	0.14
SGRQ	37.20 ± 12.85	32.20 ± 12.20	45.80 ± 10.54	34.40 ± 8.20	0.74
CAT (total score)	17.0 ± 6.32	16.20 ± 5.40	17.20 ± 4.87	14.40 ± 2.70	0.67
SF-36–General health	40.0 ± 23.18	48.00 ± 20.70	37.0 ± 14.40	39.0 ± 18.50	0.49
SF-36–Mental health	79.20 ± 17.75	82.40 ± 12.10	86.40 ± 7.26	89.60 ± 6.60	0.49
6MWT (m)	518.20 ± 10.01	502.80 ± 29.0	453.20 ± 60.27	453.0 ± 20.50	0.01*

^End of treatment between groups comparison.

*P < 0.05 indicated significant difference between groups.

6MWT: 6-minute walking test, CAT: COPD Assessment Test, FEV1: forced expiratory volume in 1 second, FVC: forced vital capacity; SGRQ: St Georges Respiratory Questionnaire, SF-36: Short-form health survey (version 2).

**Figure 3:**
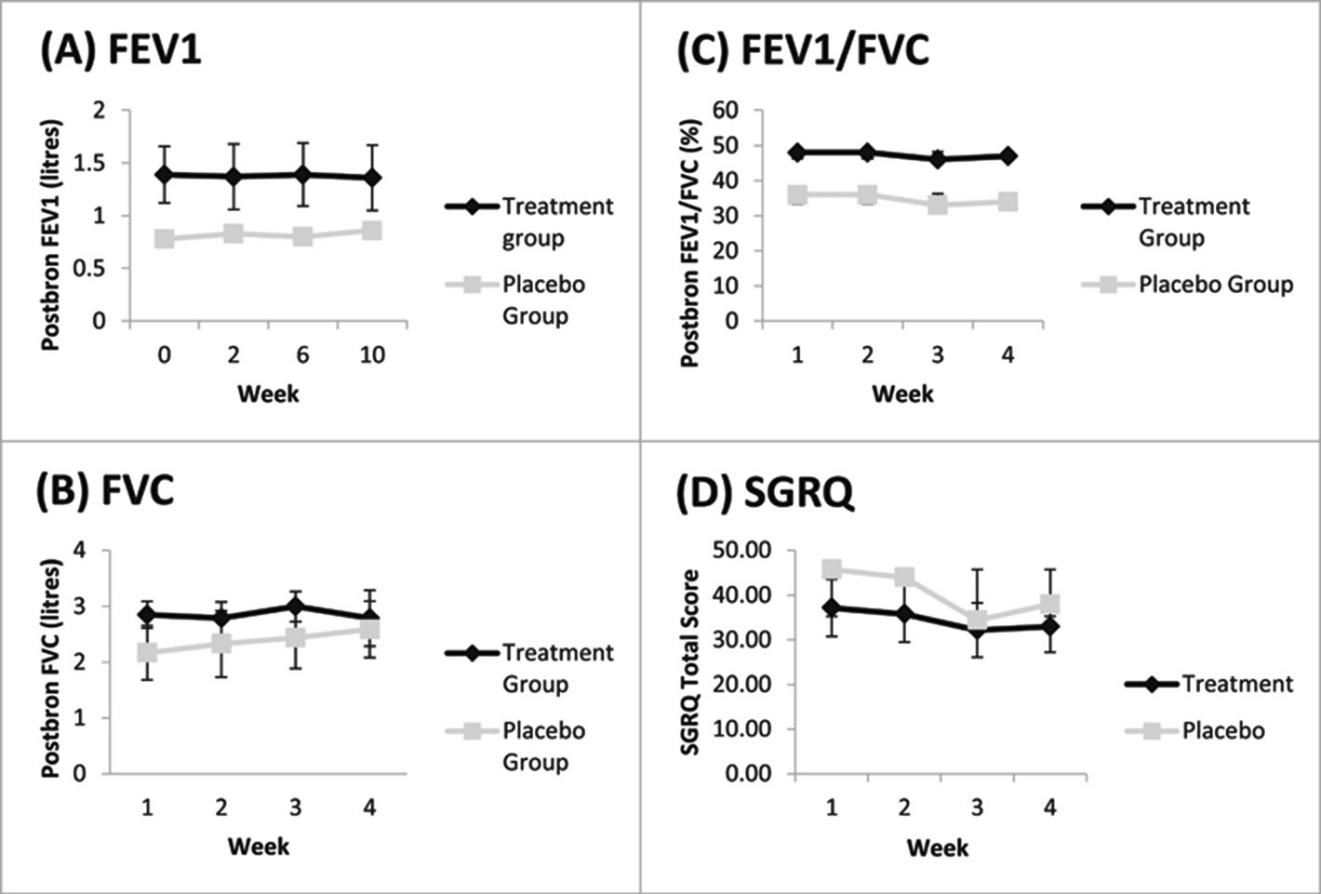
**Lung function and SGRQ results: (A) mean post-bronchodilator FEV1 in litres, (B) mean post-bronchodilator FVC in liters, (C) post-bronchodilator FEV1/FVC percentage and (D) SGRQ total score.** Error bars were standard deviations. There was no significant difference between groups at any time point.

The distance walked in six minutes at the end of treatment was not clinically important and the absolute distance walked did not change. In the *P. ginseng* group the mean distance walked at baseline was 518.2 meters and at the end of treatment it was 502.8 meters. In the placebo group, the mean distance walked at baseline and at the end of treatment did not change, 453 meters. There was also no change in mean FEV1, FVC, or FEV1/FVC in the *P. ginseng* or placebo groups (Figure 3). All participants used relief medication (salbutamol) at some stage throughout the study. On average, participants in the *P. ginseng* group used 4.82 ± 1.6 puffs per week and participants in the placebo group used 2.06 ± 1.7 puffs per week. None of the participants needed to use any other COPD-related medical resources.

There were no adverse events recorded from the study medication. One participant in the *P. ginseng* group experienced a “common cold”, with symptoms of a sore throat, cough and low-grade fever. This was not considered an exacerbation or related to the treatment. For the nine participants, blood haematology and biochemistry were unchanged after the treatment phase. The other participant showed a slight increase in white blood cell count and neutrophils. The result was not considered to be clinically important.

Potential participants were relatively easy to identify. The participants could not tell the difference between the *P. ginseng* treatment and placebo. Those given *P. ginseng* tolerated it well. Participants did not report any issues in taking the dose twice a day for both groups and did not report that the capsules tasted badly or were hard to swallow. Despite multiple outcome measures, each visit at the hospital by the participants lasted for no more than 1.5–2 hours.

## Discussion

This report presented a study protocol and the results of a pilot trial comparing *P. ginseng* with placebo for moderate to very severe COPD participants. The pilot trial was run without major issue according to the study protocol in a hospital environment in China.

The randomization process was successful and the use of opaque envelopes to conceal allocation was effective. The study procedures, including lung function, questionnaires, walking distance and blood tests were completed at all of the time points. Response rates for data collection were high; the only missing response was that of the participant who dropped out. No participants were excluded on a Chinese medicine diagnosis in the inclusion criteria.

Because of the small sample size and short study duration it was not surprising to observe no difference in outcomes. A larger trial with proper treatment duration according to the treatment principle would reveal the actual effect of *P. ginseng* in treating COPD. The planned full-scale trial protocol will be conducted over one year (6 months treatment and 6 months follow-up), similar to a current Australian trial evaluating *P. ginseng* for moderate COPD [10].

A key consideration for any full-scale trials is recruitment. In this pilot study, four out of 14 participants (28%) did not meet the inclusion criteria. Recruitment for a full-scale trial would succeed at a large hospital or multiple sites. One participant withdrew from this study. Participant withdrawals from long-term studies especially studies on COPD, are between 30% and 50% [16].

The outcome assessment in the pilot trial by only one investigator performing all tests, including spirometry using one device at the same time of day (8 a.m.–12 p.m.) might be logistically unrealistic in the full-scale trial. Therefore, standard operating procedures for all outcomes would be necessary. The definition of exacerbation severity should also be improved in a full-scale trial.

Based on the success of this pilot trial a full-scale trial has been implemented at the Guangdong Provincial Hospital of Chinese Medicine. The full-scale trial has received ethical approval and has been registered with the ANZCTR (ACTRN: 12613000382774). The sample size for that full-scale trial was calculated according to the effect size on rate of exacerbation in COPD patients using a mucolytic agent (carbocisteine) in a randomized controlled trial in China [15]. To potentially see a 30% difference between the treatment and placebo with an 80% power and a two-tailed significance level of 5%. It is estimated that a sample size of 100 participants per group would be adequate.

## Conclusions

Participant recruitment, study design, data collection and outcome measurement have been tested in a pilot trial. A full-scale trial is warranted.

## Abbreviations

6MWT: 6-Minute walking test; ANZCTR: Australian and New Zealand clinical trials registry; CAT: COPD assessment test; COPD: Chronic obstructive pulmonary disease; FER: Forced expiratory ratio; FEV1: Forced expiratory volume in one second; FVC: Forced vital capacity; GOLD: Global initiative for chronic obstructive lung disease; LOCF: Last observation carried forward; PDE-4: Phosphodiesterase-4; RCT: Randomized clinical trial; SF-36: Short-form health survey; SGRQ: St. George’s respiratory questionnaire.

## Competing interests

The authors have no competing interests.

## Authors’ contributions

CCX, LL, ALZ, FCKT, CW, LW, and ZW conceived and designed the study protocol. LW, YC, XG, ZW conducted the pilot trial. LW, YMD, JLS, and YC collected data and wrote the manuscript. All authors read and approved the final manuscript.

## Supplementary Material

Additional file 1 Human Research Ethics Committee approval. Click here for file

Additional file 2 Participant consent form. Click here for file
