# Supplementary material for: Panax ginseng therapy for chronic obstructive pulmonary disease: a clinical trial protocol and pilot study
Source: Chin Med. 2014 Aug 14;9:20. doi: 10.1186/1749-8546-9-20 (PMC4144315; doi:10.1186/1749-8546-9-20)
Supplement: Additional file 1 — Human Research Ethics Committee approval. [file 1749-8546-9-20-S1.pdf]

广东省中医院伦理委员会
Institutional Ethics Committee of Guangdong Provincial Hospital of Traditional Chinese Medicine
伦理审查批件
Approval Notice

批件号：广东省中医院伦理委员会 B2012-49-01

<table>
<tr><td>审查会议日期</td><td colspan="3">2012 年 06 月 13 日</td></tr>
<tr><td>审查会议地点</td><td colspan="3">广东省广州市大德路 111 号广东省中医院东区 12 楼会议室</td></tr>
<tr><td>临床研究批文</td><td colspan="3"></td></tr>
<tr><td>临床研究项目</td><td colspan="3">人参标准提取物治疗 COPD 中度至极重度患者：随机双盲安慰剂对照临床试验</td></tr>
<tr><td>审查文件</td><td colspan="3">1. 研究方案，版本号 1.0/20120606
2. 临床病例观察表，受试者日记卡等，版本号 1.0/20120606
3. 研究者手册，版本号 1.0/20120606
4. 课题组人员名单，课题负责人履历
5. 招募受试者的材料（包括广告）
6. 向受试者提供的研究简介，版本号 1.0/20120606
7. 向受试者提供的知情同意签字页，版本号 1.0/20120606</td></tr>
<tr><td>申办者</td><td colspan="3">广东省中医院，澳洲皇家墨尔本理工大学</td></tr>
<tr><td>临床研究单位</td><td colspan="3">广东省中医院呼吸科</td></tr>
<tr><td>主要研究者</td><td colspan="3">林琳教授</td></tr>
<tr><td>伦理审查方式</td><td colspan="3">会议审查</td></tr>
<tr><td>参会委员</td><td colspan="3">范冠杰、李艳、夏萍、许树柴、杨京华、李立凯</td></tr>
<tr><td>审查意见</td><td colspan="3">根据中华人民共和国国家药品食品监督管理局 2003 年颁布实施的《药物临床试验质量管理规范》、卫生部 2007 年颁布的《涉及人的生物医学研究伦理审查办法》，《赫尔辛基宣言》、国际医学科学组织委员会颁布的《人体生物医学研究国际道德指南》的伦理原则，经本伦理委员会审查，同意按照上述临床研究方案和上述已通过审查的文件进行<u>人参标准提取物治疗 COPD 中度至极重度患者：随机双盲安慰剂对照临床试验</u>的临床研究。</td></tr>
<tr><td>伦理委员会声明</td><td colspan="3">本批件将在各中心机构及其伦理委员会备案。如果对方案在本机构的可行性（包括研究者的资格与经验、设备与条件等）有不同意见，请及时与本伦理委员会联系。
如项目暂停/提前终止/完成临床研究，请及时通知伦理委员会。如发生严重不良事件以及影响研究风险受益比的非预期不良事件，应及时报告本伦理委员会。如临床研究方案、知情同意书的任何修改，主要研究者更换，应及时通知伦理委员会，重新审查，获得批准后执行。发现影响受试者参加研究意愿的违反方案情况应及时报告，同时，请在本批件失效日期前 1 个月提交研究进度/结题报告，以便对该项目进行跟踪审查。</td></tr>
<tr><td>批件有效期</td><td>自 2012 年 06 月 13 日起
至 2014 年 06 月 13 日止</td><td>跟踪审查频率
预计审查日期</td><td>1 年
2013 年 06 月 13 日</td></tr>
<tr><td>联系电话</td><td colspan="3">020-81887233 转 30818，联系人：何庭辉</td></tr>
<tr><td>主任委员签字</td><td colspan="3">刘军</td></tr>
<tr><td colspan="4">广东省中医院伦理委员会（盖章）</td></tr>
<tr><td colspan="4">日期：2012 年 06 月 13 日</td></tr>
</table>
