# Supplementary material for: Panax ginseng therapy for chronic obstructive pulmonary disease: a clinical trial protocol and pilot study
Source: Chin Med. 2014 Aug 14;9:20. doi: 10.1186/1749-8546-9-20 (PMC4144315; doi:10.1186/1749-8546-9-20)
Supplement: Additional file 2 — Participant consent form. [file 1749-8546-9-20-S2.pdf]

知情同意签字页

临床研究项目名称：人参标准提取物治疗COPD中度至极重度患者：随机双盲安慰剂对照临床试验

医院伦理委员会审查批件号：B2012-49-01

同意声明

我已经阅读了上述有关本研究的介绍，而且有机会就本研究与医生讨论并提出问题。我提出的所有问题都得到了满意的答复。我知道参加本研究可能产生的风险和受益。我知晓参加研究是自愿的，我确认已有充足时间对此进行考虑，而且明白：

●我可以随时向医生咨询更多的信息；

●我可以随时退出本研究而不会受到歧视或者惩罚，医疗待遇与权益不会受到影响。

我同样清楚，如果我中途退出研究，特别是由于药物的原因使我退出研究时，我若将病情变化告诉医生，完成相应的体格检查和理化检查，这将对我本人和整个研究十分有利。

如果因病情变化我需要采取任何其他的药物治疗，我会在事先征求医生的意见，或在事后如实告诉医生。我将获得一份经过签名并注明日期的知情同意书副本。

最后，我决定同意参加本项研究。

患者签名：____________________　　　　日期：________ 年 ____ 月 ____ 日

患者联系电话：____________________　　　　手机：

我确认已向患者解释了本研究的详细情况，包括其权利以及可能的受益和风险，并给予一份签署过的知情同意书副本。

医生签名：____________________　　　　日期：________ 年 ____ 月 ____ 日

医生的工作电话：______________________　　　　手机：

医院伦理委员会办公室联系电话：
